# Rasd1 is involved in white matter injury through neuron‐oligodendrocyte communication after subarachnoid hemorrhage

**DOI:** 10.1111/cns.14452

**Published:** 2023-09-22

**Authors:** Wenqiao Fu, Xudong Che, Jiahe Tan, Shizhen Cui, Yinrui Ma, Daiqi Xu, Haibo Long, Xiaolin Yang, Tangmin Wen, Zhaohui He

**Affiliations:** ^1^ Department of Neurosurgery The First Affiliated Hospital of Chongqing Medical University Chongqing China

**Keywords:** ferroptosis, neuroinflammation, Rasd1, subarachnoid hemorrhage, white matter injury

## Abstract

**Aims:**

Rasd1 has been reported to be correlated with neurotoxicity, metabolism, and rhythm, but its effect in case of subarachnoid hemorrhage (SAH) remained unclear. White matter injury (WMI) and ferroptosis participate in the early brain injury (EBI) after SAH. In this work, we have investigated whether Rasd1 can cause ferroptosis and contribute to SAH‐induced WMI.

**Methods:**

Lentivirus for Rasd1 knockdown/overexpression was administrated by intracerebroventricular (i.c.v) injection at 7 days before SAH induction. SAH grade, brain water content, short‐ and long‐term neurobehavior, Western blot, real‐time PCR, ELISA, biochemical estimation, immunofluorescence, diffusion tensor imaging (DTI), and transmission electron microscopy (TEM) were systematically performed. Additionally, genipin, a selective uncoupling protein 2(UCP2) inhibitor, was used in primary neuron and oligodendrocyte co‐cultures for further in vitro mechanistic studies.

**Results:**

Rasd1 knockdown has improved the neurobehavior, glia polarization, oxidative stress, neuroinflammation, ferroptosis, and demyelination. Conversely, Rasd1 overexpression aggravated these changes by elevating the levels of reactive oxygen species (ROS), inflammatory cytokines, MDA, free iron, and NCOA4, as well as contributing to the decrease of the levels of UCP2, GPX4, ferritin, and GSH mechanistically. According to the in vitro study, Rasd1 can induce oligodendrocyte ferroptosis through inhibiting UCP2, increasing reactive oxygen species (ROS), and activating NCOA4‐mediated ferritinophagy.

**Conclusions:**

It can be concluded that Rasd1 exerts a modulated role in oligodendrocytes ferroptosis in WMI following SAH.

## Graphical abstract

Rasd1 is mainly located in neurons after SAH. Rasd1 significantly inhibits UCP2 functions and induces ROS levels elevated. Subsequently, such changes trigger glia activation and oxidative stress and finally result in increasing NCOA4‐mediated ferritinophagy in oligodendrocytes and causes ferroptosis. Ferroptosis of oligodendrocytes contributes to white matter injury in EBI following SAH.

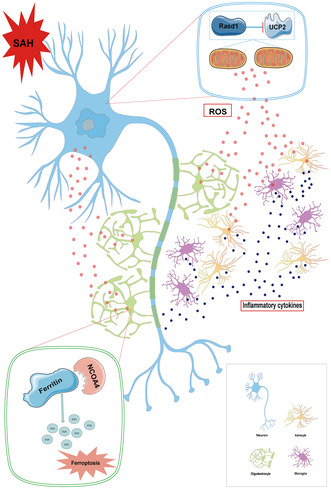

## 1 INTRODUCTION

Aneurysmal subarachnoid hemorrhage (SAH) has significant mortality rates in a short time interval, and survivors suffer from long‐term disability accompanied by physical and mental health disorders.^1^ Previous research works have primarily focused on vasospasm, delayed cerebral ischemia, and hydrocephalus. Meanwhile, white matter injury (WMI) has usually received insufficient attention.^2^ Recently, studies have reported that patients with SAH manifest extensive white matter abnormalities, which are related to cognitive impairment.^3^, ^4^ In rodent models of SAH, WMI has been observed in the early stage; such injury was characterized by a myelin loss, and white matter edema.^5^, ^6^ Therefore, developing a series of therapeutic strategies targeting WMI could contribute to improving the poor prognosis after SAH.

Oligodendrocytes (OLs) generate myelin, a mixture of proteins and lipids that can spread as compacted spirals of the biological membrane around neuronal axon fibers in the central nervous system (CNS).^7^, ^8^ Oligodendrocyte loss‐induced demyelination is a pathophysiological event among white matter injury. It can result from a variety of causes including neurodegeneration, trauma, and stroke.^9^ Current research is focused on remyelination, a process of generating new OLs from oligodendrocyte progenitor cells (OPCs); however, the role of OLs death in demyelinating disease has not been the subject of extensive studies yet, particularly in SAH.^10^ Thus, preventing the death of OLs and maintaining their normal physiological function of OLs is necessary for white matter repair.

Furthermore, ferroptosis is an iron overload‐triggered and lipid peroxidation‐driven programmed cell death, that has been widely investigated in the recent years.^11^ Multiple cellular events relevant to disease can contribute to ferroptosis, such as oxidative stress and aberrant cellular metabolism. Regulation of ferroptosis presents a great potential for the treatment of drug‐resistant tumors, traumatic injuries, and infection agents, as well as for inflammatory and neurodegenerative diseases.^12^ We and others have previously demonstrated that ferroptosis plays a pathogenic role in SAH^13^, ^14^; nevertheless, the occurrence of OLs ferroptosis following SAH remains unknown.

Besides, Rasd1 is a member of the Ras family of small G proteins, which is predominantly localized in the brain.^15^ Rasd1 plays an essential role mainly in fat metabolism, the circadian clock, anxiety disorders, and neurotoxicity.^16^, ^17^, ^18^, ^19^ It has been previously reported that down‐regulated Rasd1 and alleviated stroke injury of MCAO rats are closely connected.^20^ In fact, Rasd1 takes part in inhibiting the cAMP‐PKA‐CREB pathway in the brain.^21^ Moreover, the mitochondrial uncoupling protein 2(UCP2) was identified as a negative regulator of reactive oxygen species (ROS) and a downstream protein of the PKA‐CREB signaling pathway.^22^, ^23^ However, it is still unclear whether Rasd1 can cause brain injury by inhibiting UCP2 after SAH. Additionally, mature OLs were considered mainly to acquire iron from intracellular H‐ferritin, rather than up‐taking iron through Tf/TfR1/DMT1 pathway.^24^

In this study, we used a combination of in vivo and in vitro SAH models to investigate whether Rasd1 is involved in WMI through neuronal Rasd1/UCP2‐oligodendrocytic NCOA4/ferritin axis post‐SAH.

## 2 MATERIALS AND METHODS

### 2.1 Animals

All experiments were conducted according to the protocols proposed by the Animal Ethics Committee at the First Affiliated Hospital of Chongqing Medical University (2022‐K513). Adult male Sprague–Dawley rats weighing 180–250 g were purchased from the Chongqing Medical University Animal Experimental Center. The rats were housed at room temperature (22 ± 1°C) and constant humidity (60 ± 5%) on a 12/12 h day/night cycle, with food and water provided.

### 2.2 SAH animal model

Rats were anesthetized with 1% pentobarbital, then exposing the left internal carotid artery (ICA), puncturing the bifurcation of the left middle and the anterior cerebral artery with a 4–0 sharpened nylon wire along the ICA. The sham rats underwent the same procedure except for perforating the artery.

### 2.3 Cell culture and in vitro SAH model

Primary neurons and OLs culture were obtained as described elsewhere,^25^, ^26^ and separately incubated into the upper layer and the lower layer of Transwell for co‐culture.

According to a previous report,^27^ oxyhemoglobin (OxyHb, Sigma, USA) was used at a concentration of 10 μM to mimic the SAH environment in vitro.

### 2.4 SAH grade

According to the number of subarachnoid blood clots, the basal cistern with six segments scored a total ranging from 0 to 18, which was calculated by the accumulation of each part. Only rats with scores of at least 8 were included in this study.

### 2.5 Drug administration

#### 2.5.1 Intracerebroventricular injection in vivo

Lentiviral Rasd1 overexpression/shRNA and empty vector were purchased from GenePharma (Shanghai, China) with an injection speed of 0.5 μL/min. A total volume of 2 to 8 μL of lentivirus intraventricular injections were conducted 7 days before SAH induction.

#### 2.5.2 Lentivirus transfection and antagonist administration in vitro

Primary neurons were transfected with lentivirus using MOI 20, 40, 60, and 80. Genipin (MCE, HY‐17389, USA) was dissolved in DMSO, and the concentration in the culture media was varied where indicated (20, 40, 60, 80, and 100 μM).

### 2.6 Brain water content

To obtain wet weight, brains were immediately weighted at 24 h post‐SAH. Then, the brains were dried in an oven at 105°C for 48 h to obtain the dry weight. Finally, the brain wet‐to‐dry ratio (%) was calculated as follows: (wet weight ‐ dry weight)/(wet weight) × 100.

### 2.7 Short‐term neurological evaluation

The modified Garcia score test included spontaneous activity, four limbs movement, forelimbs outstretching, climbing, body proprioception, and vibrissae touch responses, in which the represented acute neurological dysfunction degree depended on the score.^3^, ^4^, ^5^, ^6^, ^7^, ^8^, ^9^, ^10^, ^11^, ^12^, ^13^, ^14^, ^15^, ^16^, ^17^, ^18^ Lower scores point to worse function. The beam balance test was performed on a 2.25 cm × 1 m wooden beam for 1 min, which represented rats’ walk ability depending on a score (0–4) and that a higher score indicated better driving performance and motor coordination.

### 2.8 Long‐term neurological evaluation

The learning trial was performed on the first 5 days. Rats were trained to find the platform hidden 1 cm below the water surface from four quadrants in a circle maze per day. For each trial, the session lasted for 60 s unless rats found the platform. The swimming path lengths and the time to find the platform (escape latency) were recorded accordingly. The probe trial was performed on the last day, where the rats were placed into the maze as before, but the platform was removed and the session lasted for 60 s with only one trial. The swimming speed and the time stayed in the target quadrant were recorded. All data were recorded with the ANY‐maze tracking system (Stoelting, USA).

### 2.9 Histological staining

For HE staining, brain sections firstly were dewaxed and hydrated. After being stained with hematoxylin (Beyotime, C0105s‐1, China) for 5 min and transferred into water for 5 min, the sections were shortly dipped into 1% HCl/ethanol (Beyotime, C0165S, China) for 30 s and then briefly washed 5 s with distilled water. Subsequently, they were stained with eosin (Beyotime, C0105S‐2, China) for 3 min and then washed 30 s again with distilled water. Finally, the brain sections were gradient alcohol dehydrated, xylene transparent, and then sealed.

For Prussian blue staining, brain sections were routinely dewaxed, hydrated, and washed with distilled water for 1 min and then incubated in the working solutions for 30 min and washed 5 min with distilled water; subsequently, they were dipped into Nuclear Fast Red solution for 5 min and then rinsed for 5 s. Finally, the sections were routinely dehydrated, transparent, and sealed.

Images were acquired with a Carl Zeiss AxioCam HRc camera (Zeiss, Germany) and analyzed with ImageJ software (NIH, Image J 1.46r, USA).

### 2.10 Immunofluorescence staining

Paraffin sections were incubated in 0.5% Triton solution (Beyotime, ST797) to permeabilize cell membranes for 30 min at 37°C and washed 15 min with PBS, then placed in 10 mM citrate acid buffer (pH 6.0) (Servicebio, G1201) for antigen retrieval at 100°C for 5 min and at 35°C for 15 min, subsequently cooled down to room temperature and washed with PBS again. Blocking was performed in 10% Goat Serum (Beyotime, C0265) at 37°C for 1 h, and the sections ultimately were incubated with primary antibodies to co‐staining at 4°C for at least 12 h. To bind with the primary antibodies, the sections were incubated with secondary antibodies at 37°C for 1 h. Finally, the sections were sealed by Antifade Mounting Medium using DAPI (Beyotime, P0131).

Primary neurons and OLs were fixed with 4% PFA for 30 min, then permeabilized with 0.5% Triton solution at 37°C for 1 h and washed 15 min with PBS, subsequently incubated in 10% Goat Serum (Beyotime, C0265) at 37°C for 1 h. The cells were ultimately incubated with primary antibodies and fluorescent secondary antibodies in the same procedure described above.

Images were captured using a confocal laser scanning microscope (Zeiss, LSM780) and analyzed by ImageJ software. The antibodies used were: mouse anti‐CC1 (1:200, Millipore, OP80), mouse anti‐NeuN (1:200, Abcam, ab104224), rabbit anti‐NeuN (1:200, Abcam, ab177487), rabbit anti‐ferritin (1:200, Abcam, ab75973), mouse anti‐CNPase (1:200, Abcam, ab6319), rabbit anti‐GPX4 (1:200, Abcam, ab125066), mouse anti‐Rasd1/2 (1:100, Santa Cruz, sc‐398,988), rabbit anti‐UCP2 (1:200, Proteintech, 11,081‐1‐AP), rabbit anti‐GFAP (1:200, Proteintech, 16,825‐1‐AP), rabbit anti‐IBA1 (1:200, Proteintech, 10,904‐1‐AP), rabbit anti‐NCOA4 (1:200, Affinity, DF4255), Goat anti‐rabbit Cy3 (1:100, Proteintech, SA00009‐2), and Goat anti‐mouse Alexa Fluor 488 (1:100, Abcam, ab150113).

### 2.11 Transmission electron microscopy (TEM)

Rats were anesthetized and perfused with PBS and electron microscope fixative solution (2% PFA, 2% glutaraldehyde). Then, the ipsilateral corpus callosum tissues were cut into 1 mm and fixed in 2.5% glutaraldehyde for 24 h, subsequently fixed in 1% osmium tetroxide for 1 h. After gradient dehydration and resin embedded, the sections were cut into 70 nm thick using a Leica UC7 ultramicrotome, then mounted on grids, and stained with uranyl acetate and lead citrate. Finally, myelin images were acquired using a transmission electron microscope (Hitachi, HT7800, Japan) and further analyzed by Image J software.

### 2.12 Diffusion tensor imaging (DTI)

Rats were anesthetized with isoflurane (1.5% of isoflurane mixed with oxygen), and DTI data were acquired on a 7.0 T Bruker BioSpin MRI GmbH scanner (Bruker, Germany). All data were analyzed using FSL 6.0.5.1 software (FMRIB's Software Library, www.fmrib.ox.ac.uk/fsl) and DTI studio software (Johns Hopkins School of Medicine, www.mristudio.org) to generate FA (fractional anisotropy), AD (axial diffusivity), RD (radical diffusivity), and MD (mean diffusivity). The FSL's diffusion toolkit was used to generate whole‐brain fiber tractography.

### 2.13 ROS detection

Tissue ROS levels were performed based on the manufacturer's instructions (Nanjing Jiancheng Bioengineering Institute, E004). Simply, single‐cell suspensions were incubated with 20 μM 2′,7′‐dichlorodihydrofluorescein diacetate (DCFH‐DA) at 37°C for 1 h, then washed and resuspended with PBS. Finally, the DCFH‐DA fluorescence intensity was measured by a microplate reader (Tecan, Infinite 200Pro, Switzerland).

Primary neuron ROS levels were performed depending on the manufacturer's instructions (Beyotime, S0033S). Briefly, the cells were incubated with 10 μM DCFH‐DA in PBS at 37°C for 20 min and then washed with PBS. Finally, the DCFH‐DA fluorescence intensity was measured with a fluorescence microscope (Zeiss, Germany) and analyzed with ImageJ software.

### 2.14 MDA and GSH assay

MDA and GSH contents of brain tissues and primary OLs were carried out according to the manufacturer's instructions (Nanjing Jiancheng Bioengineering Institute, A003, A006). The supernatant was incubated with a working solution for 5 min, and the absorbance was measured by a Tecan microplate reader.

### 2.15 ELISA

Brain tissue inflammatory cytokines were measured using Rat TNF alpha Uncoated ELISA Kit (Thermo Fisher Scientific, 88‐7340‐88), Rat IL‐6 Uncoated ELISA Kit (Thermo Fisher Scientific, 88‐50,625‐88), and Rat IL‐1β ELISA Kit (Lianke, EK301B/3) followed by the manufacturer's instructions.

### 2.16 Real‐time polymerase chain reaction

Total RNA was extracted from primary neurons, rats’ ipsilateral cortex and corpus callosum using TriZol Reagent (Takara, 9108). After quantified on a NanoDrop 2000c spectrophotometer (Thermo Fisher Scientific, USA) and normalized to 200 ng/μl, the RNAs were reverse transcribed into cDNA using RT Master Mix for qPCR II (MCE, HY‐K0510A) on a T100™ Thermal Cycler (BIO‐RAD, USA), then RT‐PCR was run using SYBR Green qPCR Master Mix (No ROX) (MCE, HY‐K0523) on a CFX Connect Real‐Time System (BIO‐RAD, USA). Finally, the gene expression levels were measured by the 2^−△△*C*t^ method and compared with the selected reference gene β‐Actin. All primers were purchased from Takara and the sequences were listed in Table 1.

**TABLE 1:** Primers used in real‐time PCR.

Gene	Sense primer (5′–3′)	Antisense primer (3′–5′)
β‐Actin	ACGGTCAGGTCATCACTATCG	GGCATAGAGGTCTTTACGGATG
Rasd1	CATCTGGCAATCATCCGTTTC	TGTTCTTGAGACAGGACTTGGTG
GFAP	GAAACAGAAGAGTGGTATCGGTC	ACTCAAGGTCGCAGGTCAAG
Iba‐1	TGAAGCGAATGCTGGAGAAAC	TCGGAGCCACTGGACACCT

### 2.17 Western blot analysis

Proteins were extracted from primary neurons and Ols, ipsilateral cortex and corpus callosum. The antibodies used were: rabbit anti‐ferritin (1:1000, Abcam, ab75973), mouse anti‐CNPase (1:1000, Abcam, ab6319), rabbit anti‐GPX4 (1:1000, Abcam, ab125066), mouse anti‐Rasd1/2 (1:500, Santa Cruz, sc‐398,988), mouse anti‐ARA70 (1:500, Santa Cruz, sc‐373,739), rabbit anti‐UCP2 (1:1000; Cell Signaling Technology, 89,326), mouse anti‐β‐actin (1:1000, Proteintech, 66,009‐1‐lg), anti‐rabbit secondary antibody (1:5000, Cell Signaling Technology, 7074), anti‐mouse secondary antibody (1:5000, Cell Signaling Technology, 7076).

### 2.18 Statistical analysis

All data were presented as the mean ± standard deviation (SD) and statistical evaluation was performed using GraphPad Prism 9.3.1 (GraphPad Software, USA). The data were tested for normality by using the Shapiro–Wilk test and for homogeneity of variances using the Brown–Forsythe test. Differences between the two groups were analyzed by Student's *t*‐test (two‐tailed). The differences in means among multiple groups were analyzed using one‐way ANOVA or two‐way ANOVA. *p* < 0.05 indicated a statistical significance.

## 3 RESULTS

### 3.1 Mortality and SAH grade

**FIGURE 1:**
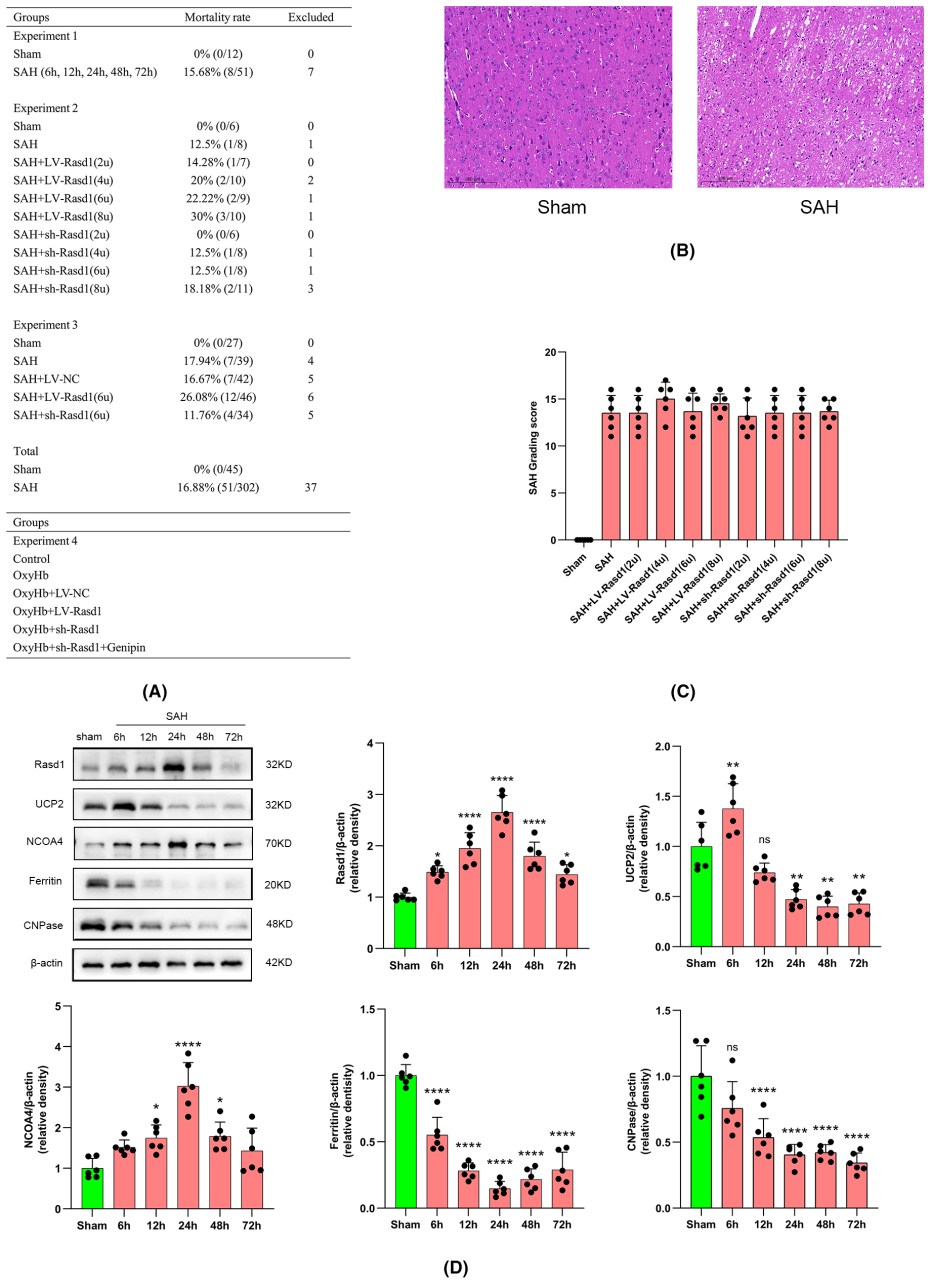
Animal experiment groups with mortality and SAH grade; cell experiment groups; temporal expression of molecules. (A) Summary of animal numbers and mortality and groups of cell experiments; (B) HE staining of Sham rat and SAH rat; (C) SAH grading scores; (D) Representative Western blot bands and relative protein expression of Rasd1, UCP2, NCOA4, ferritin and CNPase in time course experiment. Data were presented as mean ± SD, *n* = 6 per group, *^,^ **^,^ ****p < 0.05, 0.01, 0.0001 vs. Sham group, ns: not statistically significant vs. Sham group.

A total of 347 SD rats were enrolled in this study, including 45 in the sham group and 302 in the SAH group. The overall mortality was 16.88%, among 302 underwent SAH, 22 died intraoperatively and 29 died within 24 h postoperatively. Note, 37 were excluded due to SAH grade score of <8. Additionally, no obvious mortality and severity differences were noted among experimental groups (Figure 1A–C).

### 3.2 Time course of Rasd1, UCP2, NCOA4, Ferritin, and CNPase expression changes after SAH

Rasd1 and NCOA4 levels rapidly increased, peaked at 24 h, and then gradually dropped. The expression of UCP2 sharply increased and reached a maximum within 6 h and then subsequently stepwise declined. Besides, ferritin and CNPase levels consistently declined following the SAH occurrence (Figure 1D).

### 3.3 Cellular location of Rasd1

**FIGURE 2:**
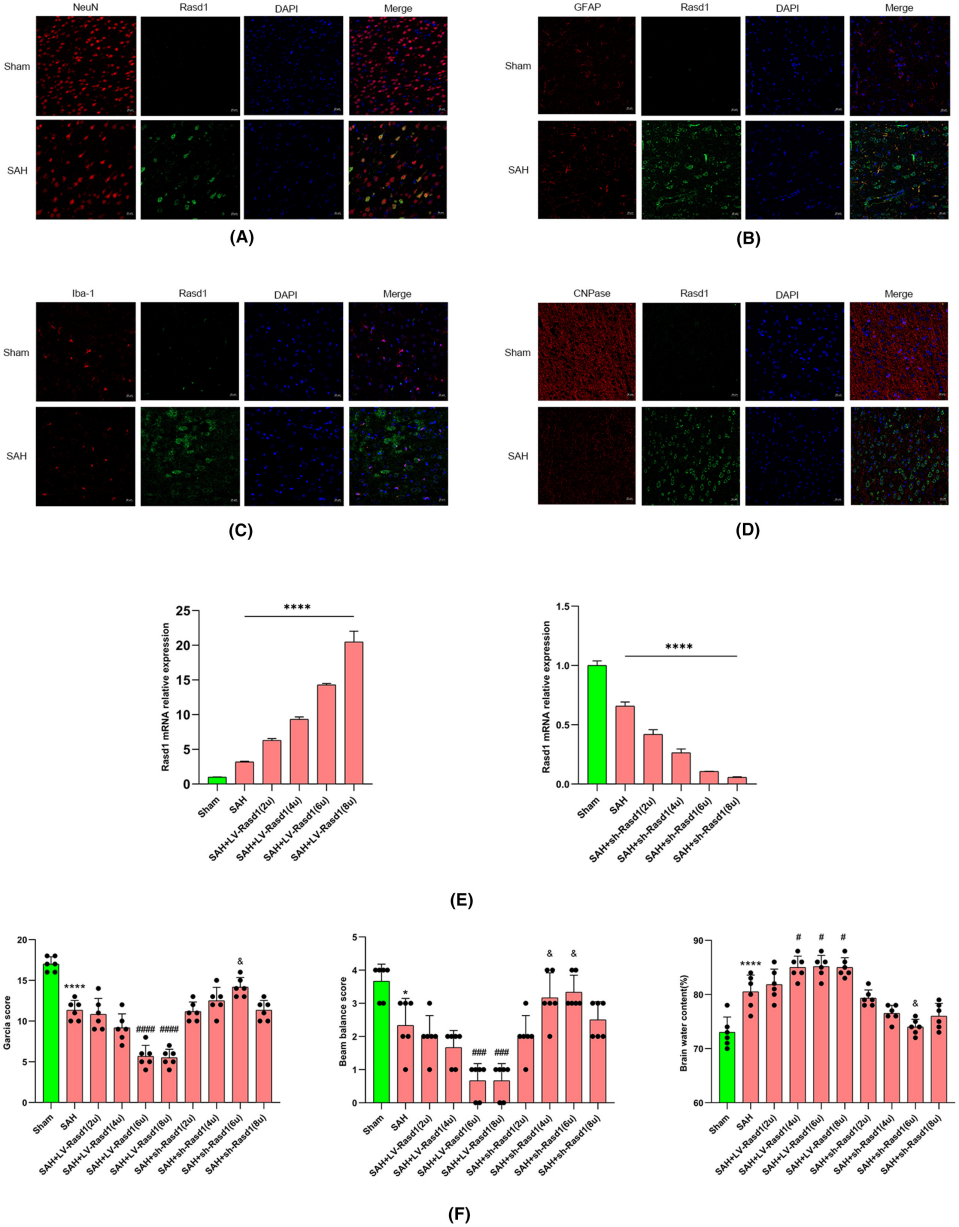
Cellular location of Rasd1; relative Rasd1 levels with neurological functions and brain water content. (A) Immunofluorescence staining of Rasd1 with neurons (NeuN, red) at 24 h after SAH, scale bar = 20 μm; (B) Immunofluorescence staining of Rasd1 with astrocytes (GFAP, red) at 24 h after SAH, scale bar = 20 μm; (C) Immunofluorescence staining of Rasd1 with microglia (Iba‐1, red) at 24 h after SAH, scale bar = 20 μm; (D) Immunofluorescence staining of Rasd1 with oligodendrocytes (CNPase, red) at 24 h after SAH, scale bar = 20 μm; (E) Relative mRNA expression of Rasd1; (F) Garcia score, Beam balance score and Brain water content (%) of all SAH groups. Data were presented as mean ± SD, *n* = 6 per group, *^,^ ****p < 0.05, 0.0001 vs. Sham group, ^#, ###, ####^ *p* < 0.05, 0.001, 0.0001 vs. SAH + LV‐Rasd1(2u) group, ^&^ *p* < 0.05 vs. SAH + sh‐Rasd1(2u) group.

Double immunofluorescence staining and co‐localization analysis showed the notable expression of Rasd1, which was significantly increased in neurons, astrocytes, microglia, and oligodendrocytes after SAH. Worth to be mentioned, neurons accounted for the majority of Rasd1‐positive cells compared with other cells (Figure 2A–D).

### 3.4 The role of Rasd1 in the neurological outcome and brain water content after SAH

We genetically upregulated and downregulated Rasd1 via lentiviral transduction by intracerebroventricular injections. Real‐time PCR demonstrated that the mRNA level of Rasd1 had raised proportionally with the gradual gradient increase of the LV‐Rasd1 dose, and vice versa (Figure 2E). Besides, compared with the sham group, SAH group rats showed remarkable neurological impairments under evaluation of modified Garcia, beam balance test, and cerebral edema. The results suggested severity of the neurological deficits that reached a maximum degree after rats were subjected to intermediate (6u) and high doses (8u) of LV‐Rasd1. It was noticed that the administration of 8u LV‐Rasd1 was accompanied by the highest mortality. Simultaneously, intraventricular injection of 6u sh‐Rasd1 attenuated the neurological dysfunction, mostly in all lentiviral‐transfected groups (Figure 2F).

### 3.5 The effect of Rasd1 on long‐term neurobehavior after SAH

**FIGURE 3:**
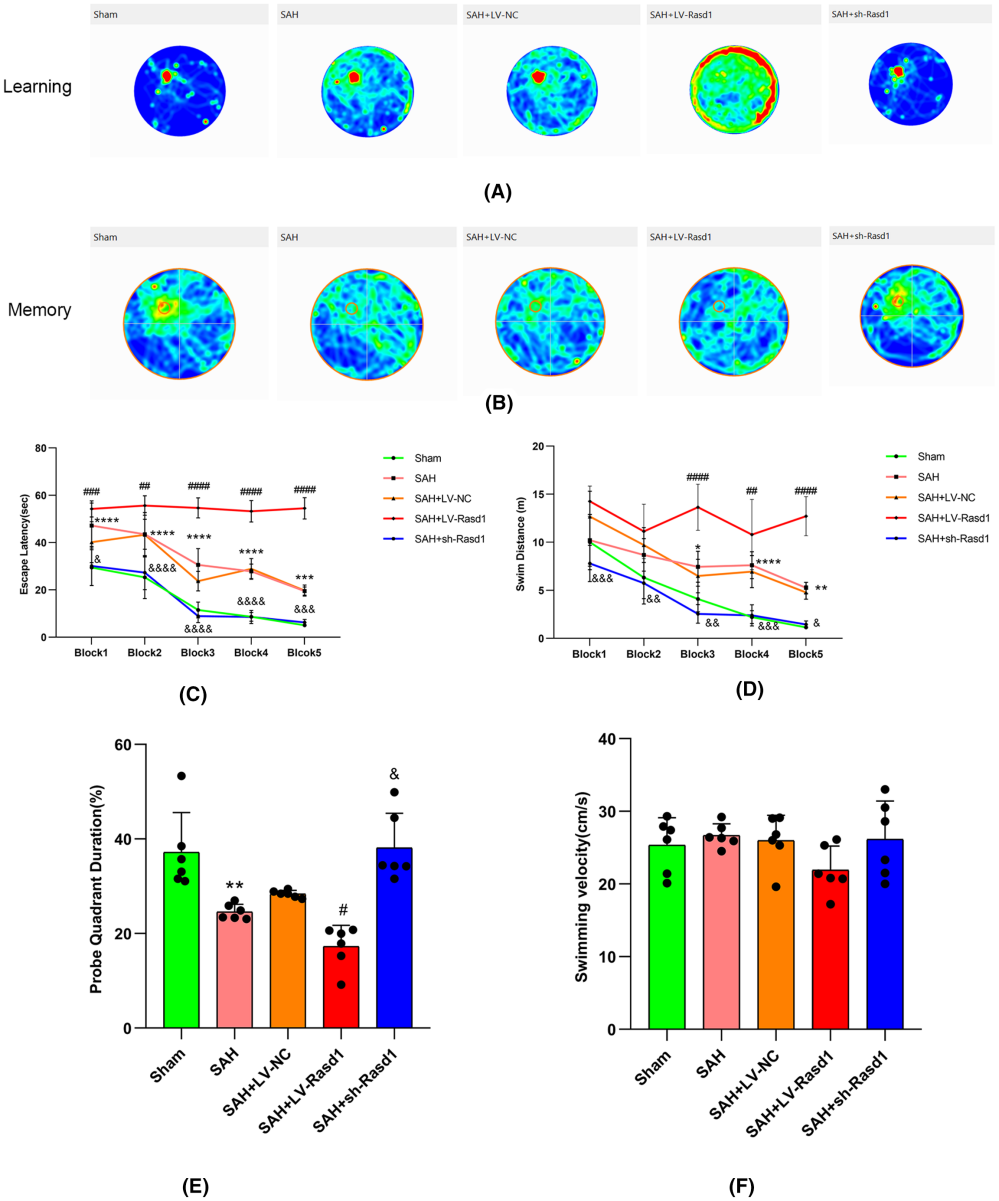
The effect of Rasd1 on long‐term neurobehavior after SAH. (A) Representative heat map of movement trajectories in spatial learning test; (B) Representative heat map of movement trajectories in probe trial; (C) Escape latency in spatial learning test; (D) Swim distance in spatial learning test; (E) The target quadrant duration in the probe trial; (F) Swimming velocities of different groups in the probe trial. Data were presented as mean ± SD, *n* = 6 per group, **^,^ ***^,^ ****p < 0.01, 0.001, 0.0001 vs. Sham group, ^#, ##, ###, ####^ *p* < 0.05, 0.01, 0.001, 0.0001 vs. SAH + LV‐NC group, ^&, &&, &&&, &&&&^ *p* < 0.05, 0.01, 0.001, 0.0001vs. SAH + LV‐NC group.

Rats were assessed by a widely used spatial learning and memory test of Morris water maze (MWM) from day 21 to 25 post‐SAH induction. During the acquisition trials, the swimming velocity did not differ significantly within the different groups. SAH group rats compared with sham groups apparently manifested a spatial learning disability of prolonged escape latency in the blocks 1, 2, 3, 4, and, 5, and a larger swimming distance in the blocks 3, 4, and, 5. Moreover, SAH + LV‐Rasd1 rats, compared with SAH + LV‐NC rats, have spent more time in the blocks 1, 2, 3, 4, and 5 and swam a longer distance in the blocks 3, 4, and, 5, to find the platform beneath the water surface. With respect to the rats receiving SAH + sh‐Rasd1, they had a dramatically shortened escape latency in the blocks 1, 2, 3, 4, and, 5, and shorter swimming distance in the blocks 3, 4, and, 5 (Figure 3A,C,D). During the probe trials undertaken, compared with the sham rats, the SAH rats exhibited memory loss as a result of more time spent in the target quadrant. Besides, rats of SAH + LV‐Rasd1 stayed for a longer duration in the target quadrant than that of LV‐Rasd1 application, while the administration of sh‐Rasd1 showed reverse results (Figure 3B,E). All rats had no obvious differences in swimming velocity during the experiment (Figure 3F).

### 3.6 Rasd1 is involved in regulating glia activation after SAH

**FIGURE 4:**
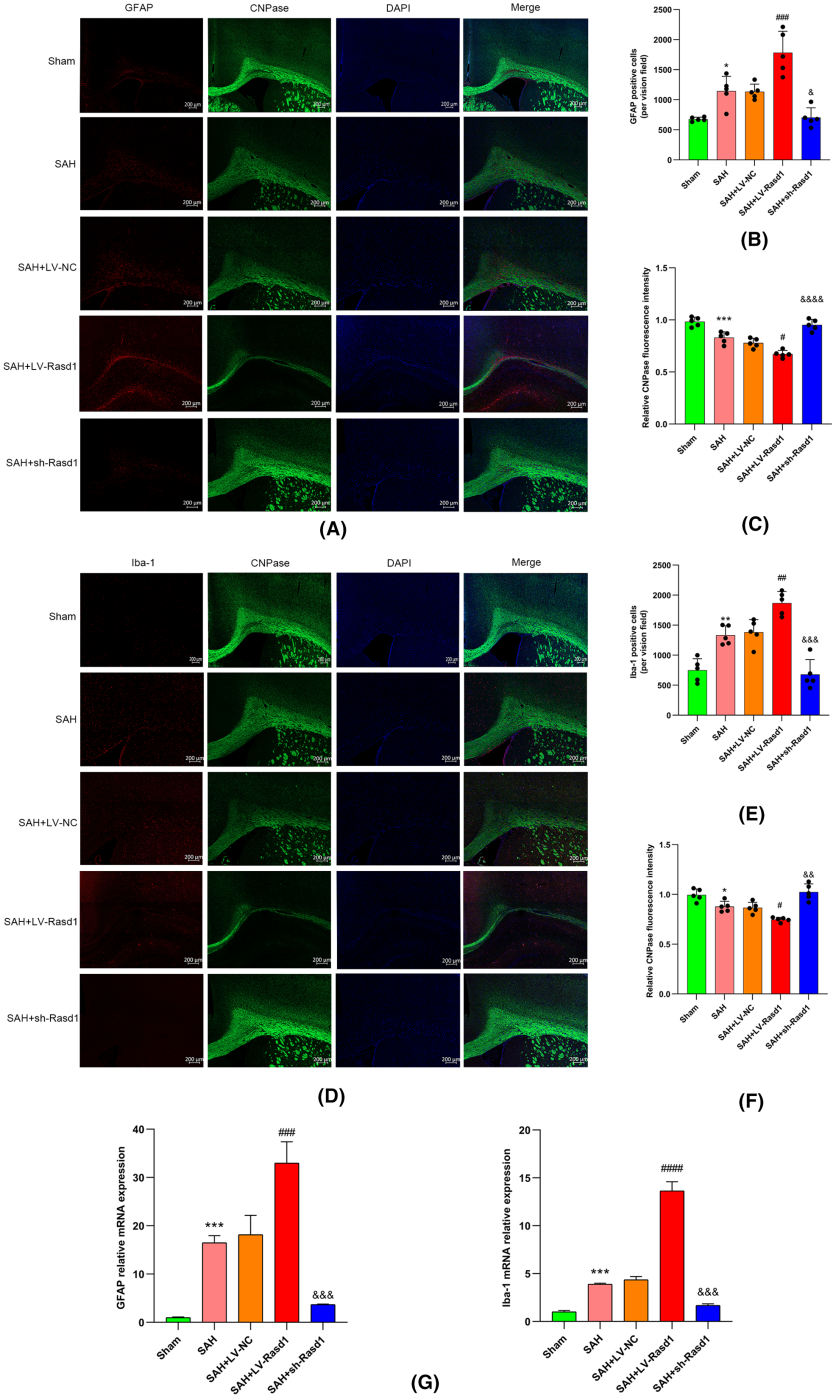
Rasd1 involved in regulating glia activation after SAH in the way of serial section. (A) Double immunostaining of GFAP and CNPase at 24 h after SAH, scale bar = 200 μm; (B) GFAP^+^ cells amount per vision field; (C) Relative fluorescence intensity of CNPase; (D) Double immunostaining of Iba‐1 and CNPase at 24 h after SAH, scale bar = 200 μm; (E) Iba‐1^+^ cells amount per vision field; (F) Relative fluorescence intensity of CNPase; (G) Relative mRNA expression of GFAP and Iba‐1 at 24 h after SAH. Data were presented as mean ± SD, *n* = 5 per group, *^,^ **^,^ ***^,^ ****p < 0.05, 0.01, 0.001, 0.0001 vs. Sham group, ^#, ###, ####^ *p* < 0.05, 0.001, 0.0001 vs. SAH + LV‐NC group, ^&, &&, &&&, &&&&^ *p* < 0.05, 0.01, 0.001, 0.0001 vs. SAH + LV‐NC group.

Oligodendrocytes (CNPase) respectively immuno‐co‐stained with astrocytes (GFAP), microglia (Iba‐1) in the left cerebral cortex, and corpus callosum in the way of serial section. Meanwhile, GFAP and Iba‐1 mRNA levels were detected by real‐time PCR. Data from staining revealed that SAH led to an increase of GFAP‐positive and Iba‐1‐positive cells compared with the sham group. In addition, with respect to the SAH + LV‐NC group, the number of astrocytes and microglia from SAH + LV‐Rasd1 was larger, while the number of SAH + sh‐Rasd1 group glia cells became reduced to few cells. The CNPase fluorescence intensity variation showed an opposite trend with those results observed above (Figure 4A–F). Real‐time PCR results showed GFAP and Iba‐1 transcript levels from diverse groups changed in agreement with the variation trends confirmed earlier by IF staining (Figure 4G).

### 3.7 Rasd1 participated in neuroinflammation and oxidative stress via modulating neuronal UCP2 after SAH

**FIGURE 5:**
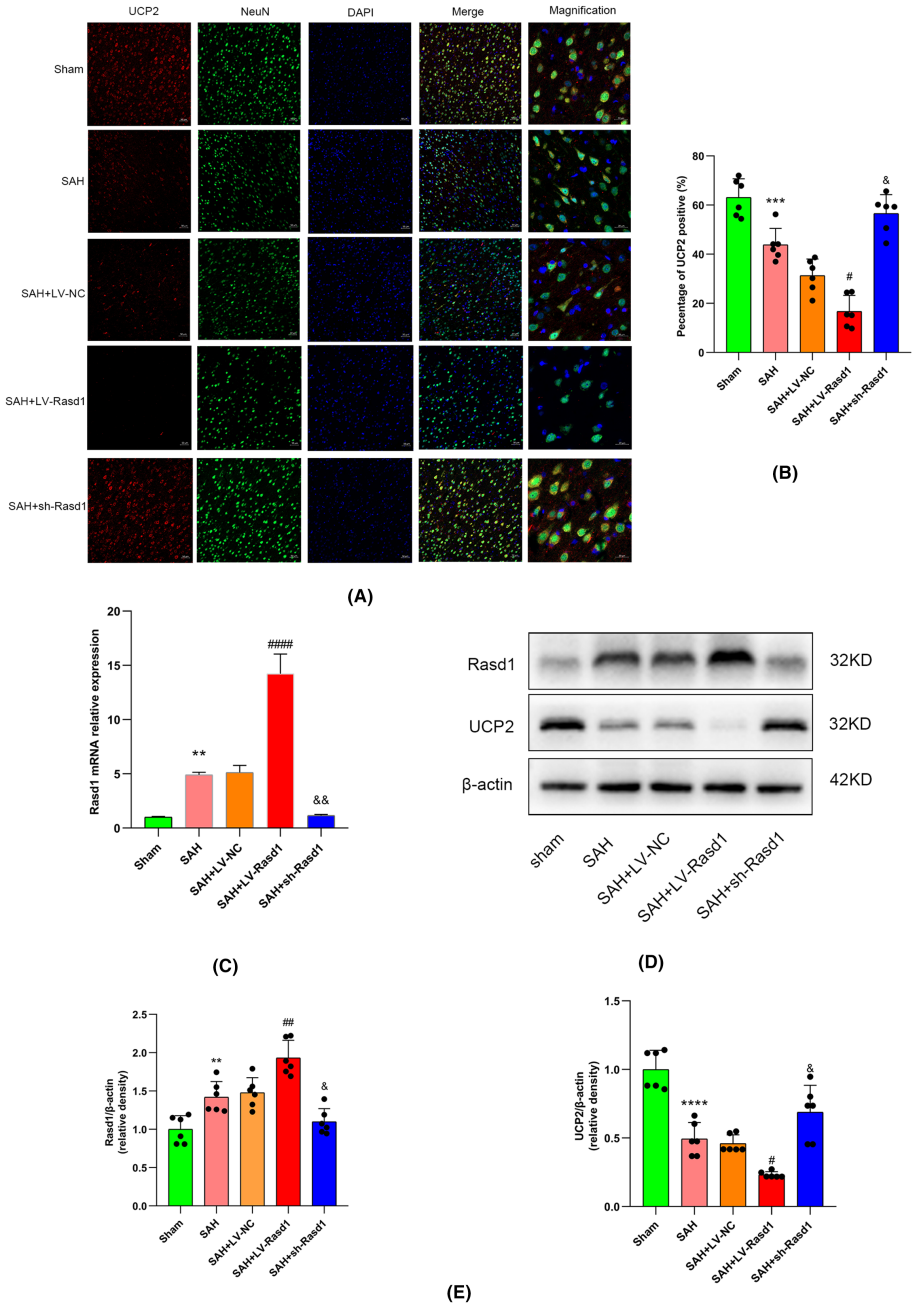
The effect of Rasd1 on UCP2 inhibition. (A) Immunofluorescence staining of UCP2 (red) with neurons (NeuN, green) at 24 h after SAH, scale bar = 50 μm or 20 μm; (B) Percentage of UCP2 positive cells; (C) Relative mRNA expression of Rasd1 at 24 h after SAH; (D) Representative Western blot bands of Rasd1 and UCP2 at 24 h after SAH; (E) Relative protein expression of Rasd1 and UCP2 at 24 h after SAH. Data were presented as mean ± SD, *n* = 6 per group, **^,^ ***^,^ ****p < 0.01, 0.001, 0.0001 vs. Sham group, ^#, ##, ####^ *p* < 0.05, 0.01, 0.0001 vs. SAH + LV‐NC group, ^&, &&^ *p* < 0.05, 0.01 vs. SAH + LV‐NC group.

**FIGURE 6:**
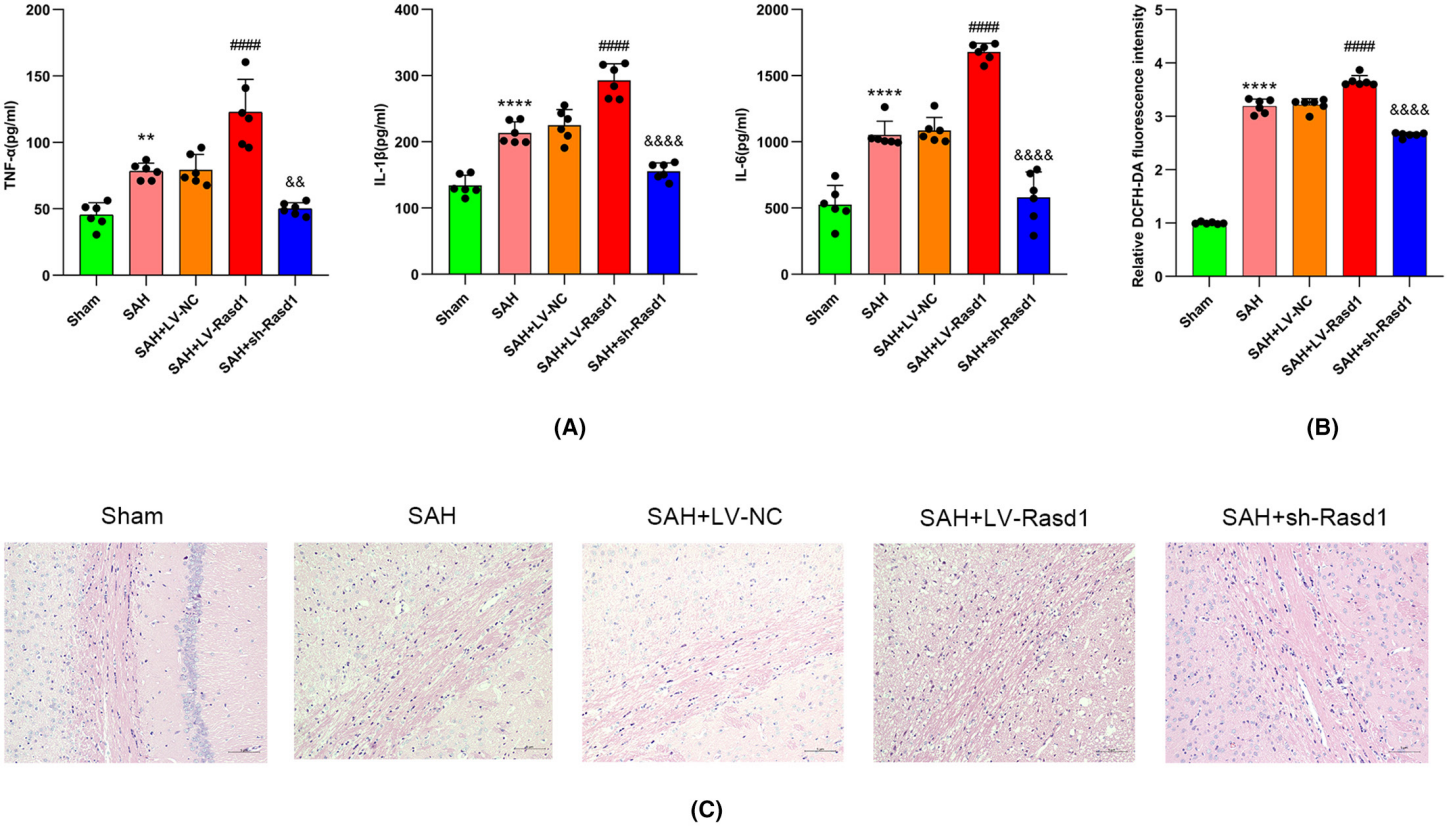
The role of Rasd1 on neuroinflammation and oxidative stress after SAH. (A) Determination of TNF‐α, IL‐1β, and IL‐6 levels by ELISA; (B) Relative DCFH‐DA fluorescence intensity; (C) HE staining of the corpus callosum at 24 h after SAH, scale bar = 5 μm. Data were presented as mean ± SD, *n* = 6 per group, **^,^ ****p < 0.01, 0.0001 vs. Sham group, ^####^ *p* < 0.05, 0.0001 vs. SAH + LV‐NC group, ^&&, &&&&^ *p* < 0.01, 0.0001vs. SAH + LV‐NC group.

UCP2 has been reported to reduce ROS production by regulating the mitochondrial electron transport chain,^28^ and Rasd1 had an inhibitory effect on the PKA‐CREB pathway. Therefore, in order to examine whether a direct connection existed between Rasd1 and UCP2 and explore the role of Rasd1 in inflammatory response, and oxidative stress, ELISA, ROS assay, HE staining, Western blot, and IF staining were performed. Western blot and IF staining analysis showed that SAH led to the decline of UCP2 protein levels. Subsequently, the Rasd1 overexpression evidently decreased the UCP2 expression, whereas sh‐Rasd1 injection effectively reversed the inhibition (Figure 5). Data from ROS assay and ELISA demonstrated that SAH has enhanced the ROS production and inflammatory cytokines (TNF‐α, IL‐1β, and IL‐6) release, as well as the overexpressed Rasd1 derived a promotion on ROS accumulation and inflammatory factors secretion, while downregulated Rasd1 abolished the effect (Figure 6A,B). Data from HE staining indicated SAH resulted in ipsilateral cortex and corpus callosum tissue edema with cell death, inflammatory cell infiltration, and fiber sparseness. LV‐Rasd1 transfection significantly exacerbated these pathological changes; meanwhile, sh‐Rasd1 prevented the effect caused by SAH (Figure 6C).

### 3.8 The association of Rasd1 in white matter injury after SAH

**FIGURE 7:**
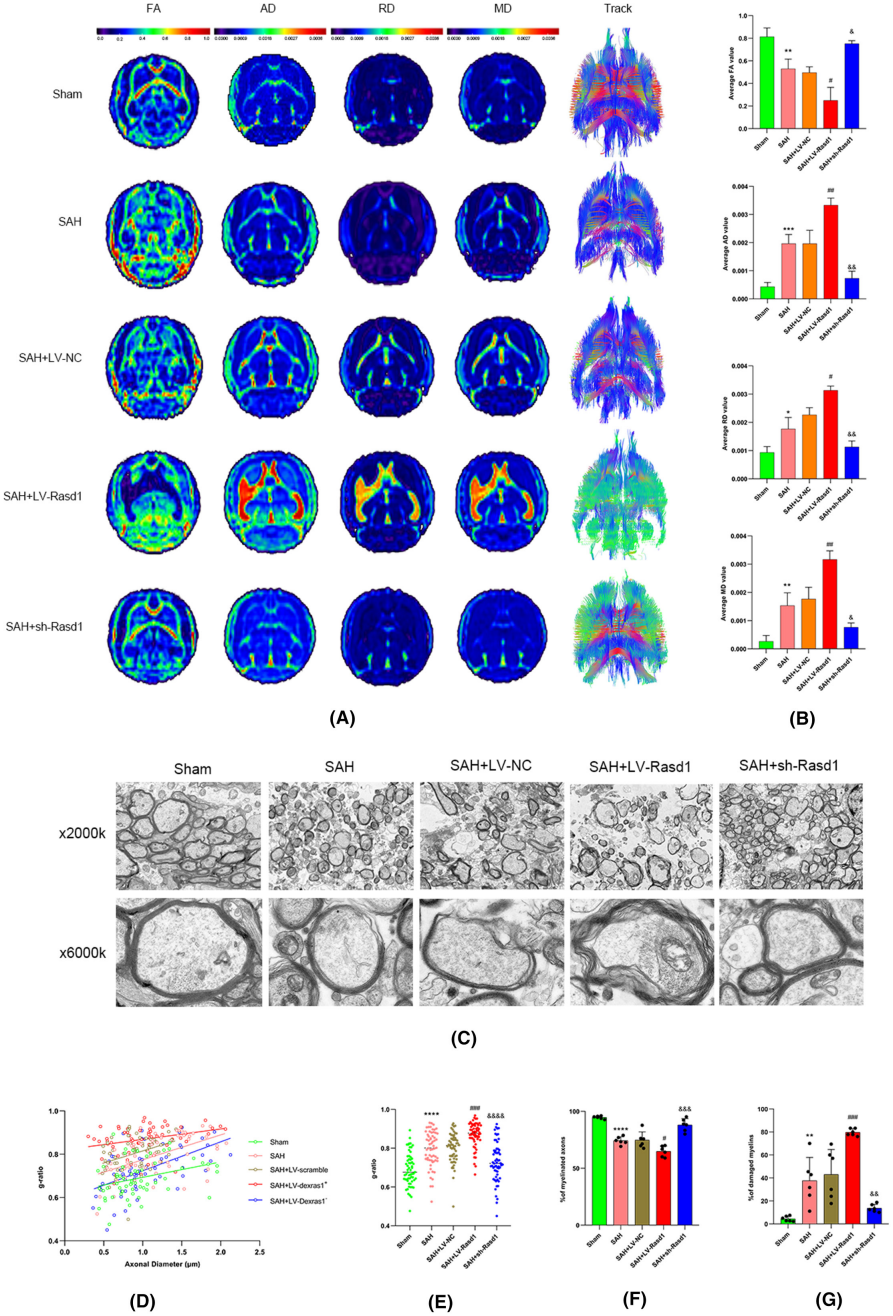
The effect of Rasd1 in white matter injury after SAH. (A) The diffusion tensor imaging of rats in indifferent groups, AD, axial diffusivity; FA, fractional anisotropy; MD, mean diffusivity; RD, radical diffusivity; (B) Quantification of average FA, AD, RD, and MD value; (C) Electron micrographs of myelin, scale bar = 5 μm upper panel, 500 μm down panel; (D) Scatter plot of g‐ratio values of myelin with axon diameter among all groups after SAH; (E) Mean g‐ratio values of myelin among all groups after SAH; (F) Percentage of myelinated axons; (G) Percentage of damaged myelin. Data were presented as mean ± SD, *n* = 3 per group, *^,^ **^,^ ***^,^ ****p < 0.05, 0.01, 0.001, 0.0001 vs. Sham group, ^#, ##, ###, ####^ *p* < 0.05, 0.01, 0.001, 0.0001 vs. SAH + LV‐NC group, ^&, &&, &&&, &&&&^ *p* < 0.05, 0.01, 0.001, 0.0001vs. SAH + LV‐NC group.

The association between Rasd1 and whiter matter injury was assessed using DTI, TEM, and g‐ratio from macrostructural and microstructural perspectives separately. From a macrostructural perspective of white matter, WMI was assessed by DTI metrics (FA, MD, AD, and RD) and an entire view of white matter fiber tracts through reconstruction. On the one hand, compared with the sham group, the SAH group showed lower FA values, suggesting a worse white matter integrity and sparser fiber bundles. While the LV‐Rasd1 treatment further decreased the FA values, however, the sh‐Rasd1 treatment had an opposite effect, when compared with the SAH + LV‐NC group. On the other hand, in contrast to artery unpunctured rats, SAH rats exhibited higher MD, AD, and RD values, indicating greater diffusion of water molecules, which also means myelination is impaired. The LV‐Rasd1 administration affect the myelin to become thinner, however, under the sh‐Rasd1 administration, the myelin became thicker when in contrast to the control group (SAH + LV‐NC) rats. Meanwhile, the reconstructed corpus callosum fiber tract analysis showed that SAH had caused decreased fiber density; hence interrupting continuity and disordered orientation of the fibers. In contrast to the control group (SAH + LV‐NC), the LV‐Rasd1 transfection substantially exacerbated the above changes, while sh‐Rasd1 prominently attenuated them (Figure 7A,B). From a microstructural perspective of myelin, we used TEM images to analyze the myelin morphological changes. Myelin swelling, sparse or delamination, and even break were observed in the SAH group, LV‐Rasd1 injection further dramatically exacerbated myelin impairment, while sh‐Rasd1 injection notably attenuated this impairment. Similar to previous results, the SAH group showed a higher g‐ratio than the sham group, the SAH + LV‐Rasd1 group achieved the highest g‐ratio while SAH + sh‐Rasd1 restored SAH‐mediated high myelin g‐ratio (Figure 7F,G).

### 3.9 Rasd1 via neurons indirectly induces oligodendrocytes ferroptosis in the form of NCOA4‐mediated ferritinophagy

**FIGURE 8:**
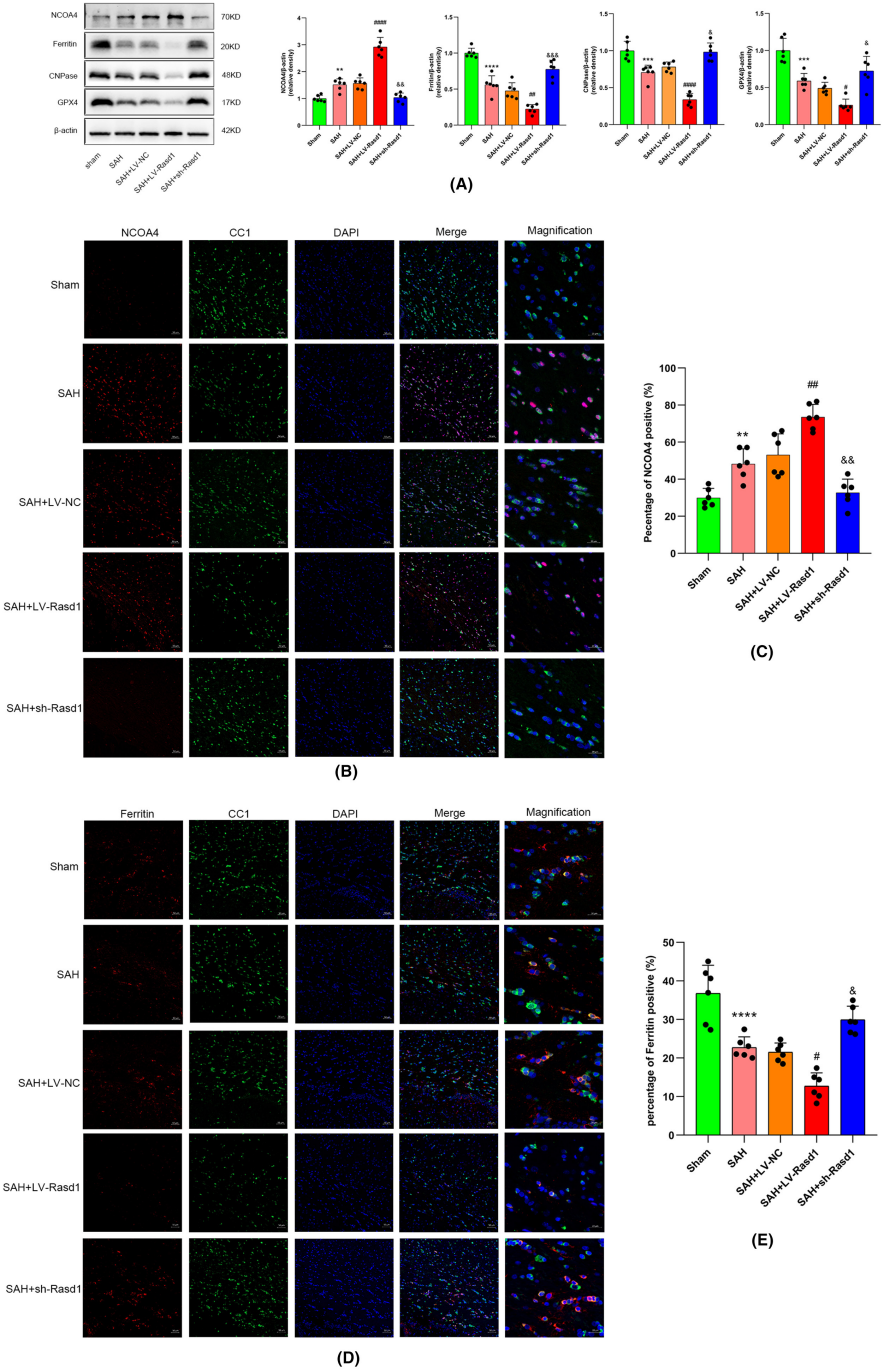
Rasd1 increases the level of NCOA4‐mediated ferritinophagy in oligodendrocytes. (A) Representative Western blot bands and relative protein expression of NCOA4, ferritin, CNPase and GPX4 at 24 h after SAH; (b) Immunofluorescence staining of NCOA4 (red) with oligodendrocytes (CC1, green) at 24 h after SAH, scale bar = 50 μm or 20 μm; (c) Percentage of NCOA4‐positive cells; (D) Immunofluorescence staining of ferritin (red) with oligodendrocytes (CC1, green) at 24 h after SAH, scale bar = 50 μm or 20 μm; (E) Percentage of ferritin‐positive cells. Data were presented as mean ± SD, *n* = 6 per group, **^,^ ***^,^ ****p < 0.01, 0.001, 0.0001 vs. Sham group, ^#, ##, ####^ *p* < 0.05, 0.01, 0.0001 vs. SAH + LV‐NC group, ^&, &&, &&&^ *p* < 0.05, 0.01, 0.001 vs. SAH + LV‐NC group.

**FIGURE 9:**
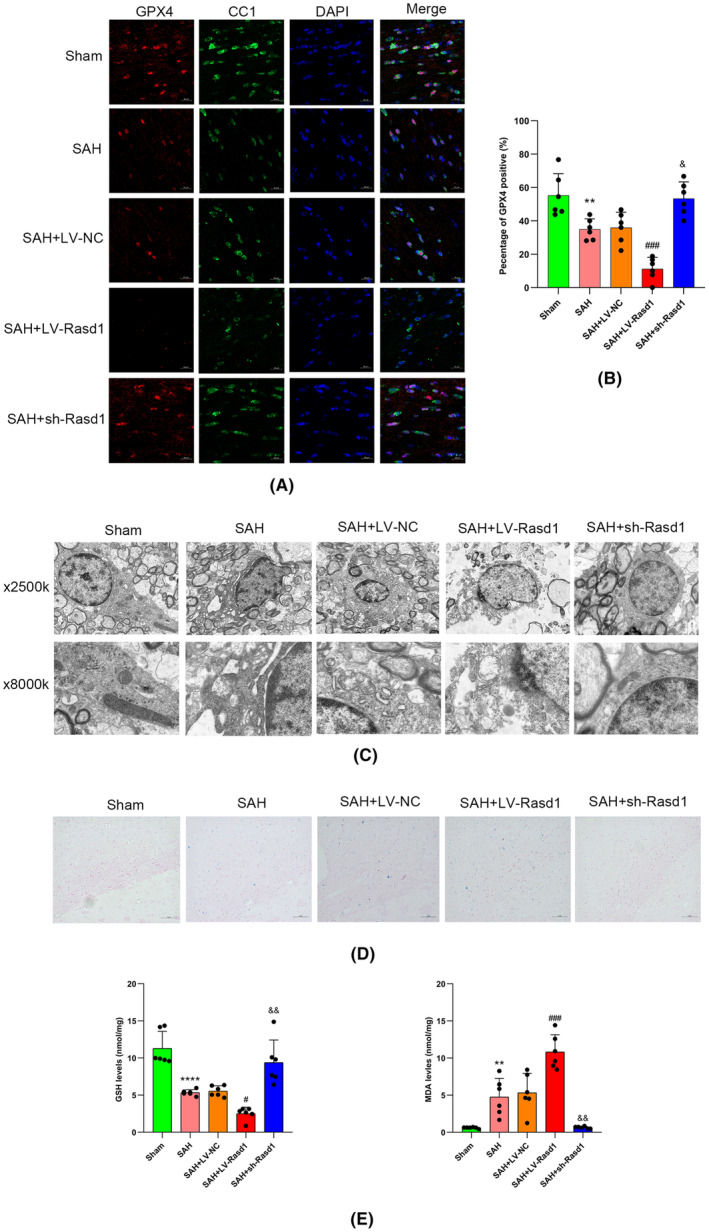
Rasd1 induces oligodendrocytes ferroptosis and increases lipid peroxidation levels. (A) Immunofluorescence staining of GPX4 (red) with oligodendrocytes (CC1, green) at 24 h after SAH, scale bar = 20 μm; (B) Percentage of GPX4 positive cells; (C) Electron micrographs of oligodendrocytes mitochondria, scale bar = 2 μm upper panel, 1um down panel; (D) Prussian blue staining, scale bar = 5 μm; (E) Determination of GSH and MDA level by ELISA. Data were presented as mean ± SD, *n* = 6 per group, **^,^ ****p < 0.01, 0.0001 vs. Sham group, ^#, ###^ *p* < 0.05, 0.001 vs. SAH + LV‐NC group, ^&, &&^ *p* < 0.05, 0.01 vs. SAH + LV‐NC group.

Both Western blot and IF staining revealed that the levels of ferritin, GPX4, and CNPase were decreased, and the level of NCOA4 was increased after SAH. It can be deduced that Rasd1 overexpression enhanced NCOA4 expression and suppressed ferritin, GPX4, and CNPase expression, while Rasd1 knockdown manifested an adverse variation trend (Figures 8 and 9A,B). Prussian blue staining showed iron deposited in the ipsilateral cortex and corpus callosum after SAH. Subsequently, the Rasd1 overexpression expanded iron deposition scope, whereas Rasd1 knockdown terminated iron deposition caused by SAH (Figure 9D). TEM analysis showed OLs mitochondrial changes after SAH as follows: reduced mitochondrial numbers, smaller mitochondrial volume, mitochondrial cristae reduced even disappeared, outer mitochondrial membrane ruptured. We observed that LV‐Rasd1 transfection further significantly aggravated OLs mitochondrial ferroptosis‐like morphological deficits, while sh‐Rasd1 injection effectively prevented the development of these changes after SAH (Figure 9C). Hence, SAH increased the level of lipid peroxidation characterized by elevated MDA levels and reduced GSH levels. Similar to the above results, LV‐Rasd1 transfection aggravated the lipid peroxidation level, while sh‐Rasd1 attenuated it (Figure 9E).

### 3.10 Rasd1 increases neurons’ oxidative stress through blocking UCP2 activation in vitro

**FIGURE 10:**
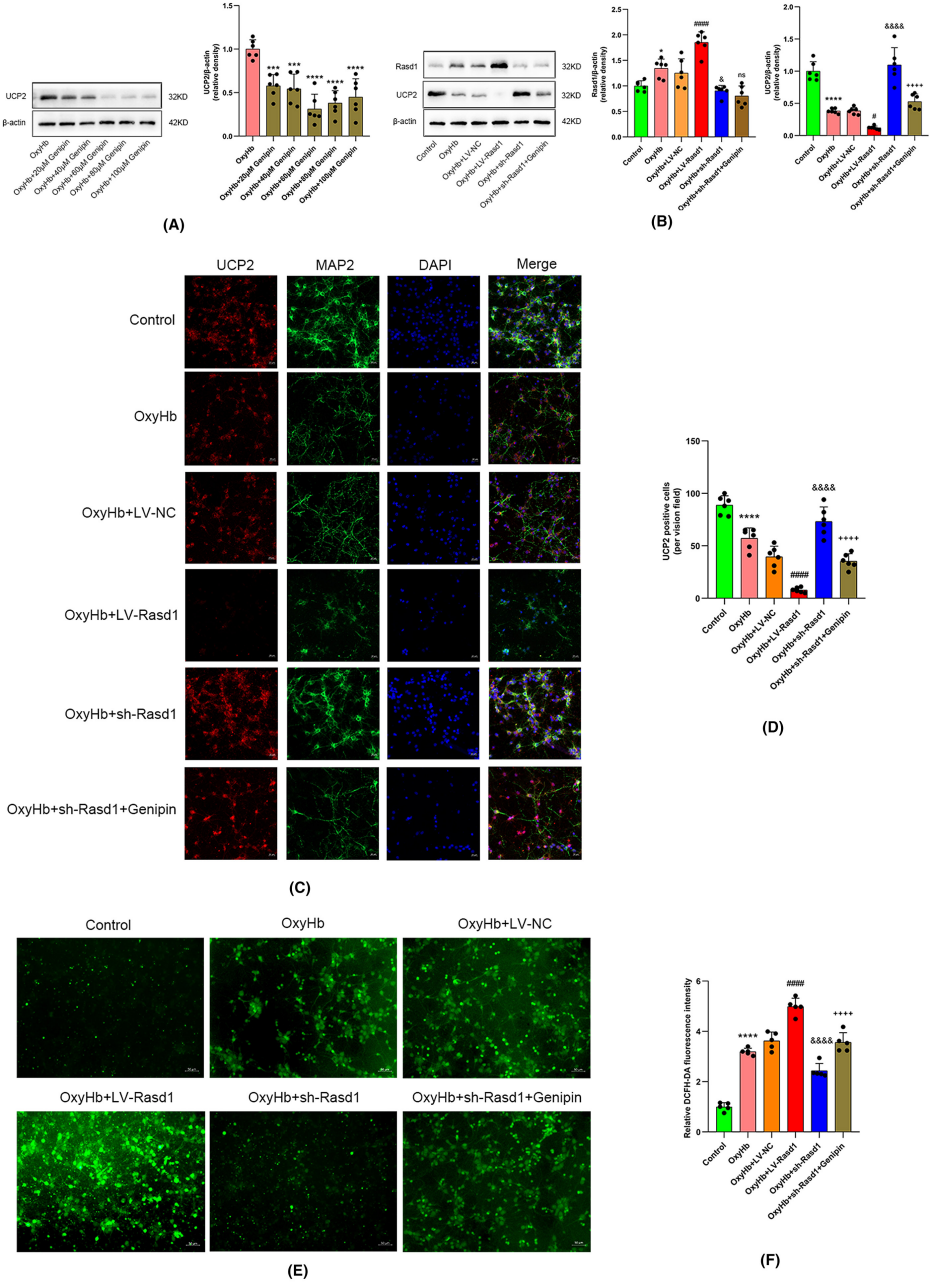
Rasd1 increases neurons’ oxidative stress through blocking UCP2 activation in vitro. (A) Representative Western blot bands and relative protein expression of UCP2 in gradient genipin concentration groups, *n* = 6 per group; (B) Representative Western blot bands and relative protein expression of Rasd1 and UCP2, *n* = 6 per group; (C) Immunofluorescence staining of UCP2 (red) with neurons (MAP2, green), scale bar = 20 μm; (D) UCP2‐positive cells (per vision field), *n* = 6 per group; (E) ROS detection by fluorescence staining, scale bar = 50 μm; (F) Relative DCFH‐DA fluorescence intensity for intracellular ROS, *n* = 5 per group. Data were presented as mean ± SD, *^,^ ***^,^ ****p < 0.05, 0.001, 0.0001 vs. Control group, ^#, ####^ *p* < 0.05, 0.0001 vs. OxyHb + LV‐NC group, ^&, &&&&^ *p* < 0.05, 0.0001vs. OxyHb + LV‐NC group, ^++++^ *p* < 0.0001 vs. OxyHb + sh‐Rasd1 group, ns, not statistically significant vs. OxyHb + sh‐Rasd1 group.

**FIGURE 11:**
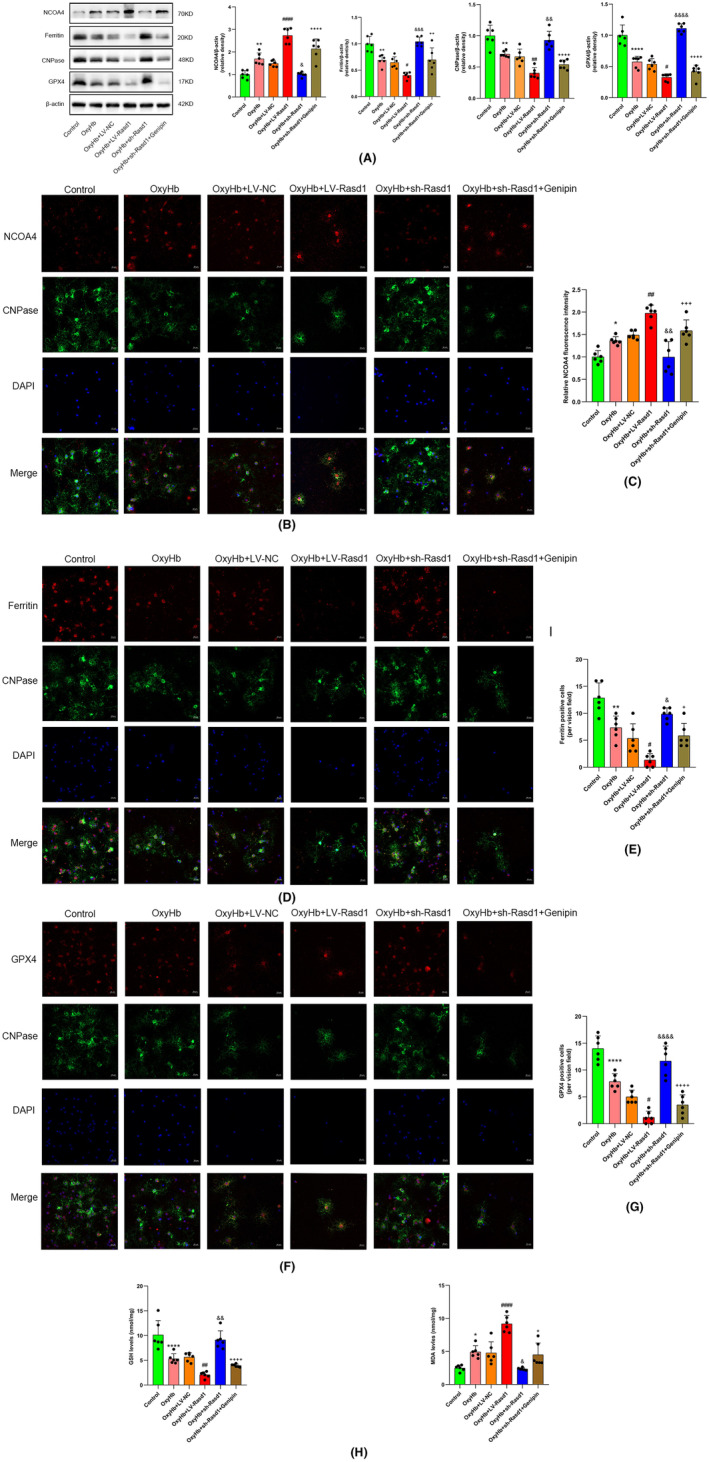
Rasd1 indirectly causes NCOA4‐mediated induced oligodendrocytes ferroptosis in vitro. (A) Representative Western blot bands and relative protein expression of NCOA4, ferritin, CNPase and GPX4; (B) Immunofluorescence staining of NCOA4 (red) with oligodendrocytes (CNPase, green), scale bar = 20 μm; (C) Relative fluorescence intensity of NCOA4; (D) Immunofluorescence staining of ferritin (red) with oligodendrocytes (CNPase, green), scale bar = 20 μm; (E) ferritin‐positive cells (per vision field); (F) Immunofluorescence staining of GPX4 (red) with oligodendrocytes (CNPase, green), scale bar = 20 μm; (G) GPX4‐positive cells (per vision field); (H) Determination of GSH and MDA level in oligodendrocytes by ELISA. Data were presented as mean ± SD, *n* = 6 per group, *^,^ **^,^ ****p < 0.05, 0.01, 0.0001 vs. Control group, ^#, ##, ####^ *p* < 0.05, 0.01, 0.0001 vs. OxyHb + LV‐NC group, ^&, &&, &&&, &&&&^ *p* < 0.05, 0.01, 0.001, 0.0001 vs. OxyHb + LV‐NC group, ^+, ++, +++, ++++^ *p* < 0.05, 0.01, 0.001, 0.0001 vs. OxyHb + sh‐Rasd1 group, ns: not statistically significant vs. OxyHb + sh‐Rasd1 group.

Western blot results showed the optimal inhibitory UCP2 affect neurons achieved when genipin concentration was 60 μM in OxyHb‐treated primary neurons (Figure 10A). As expected, OxyHb treatment declined UCP2 expression, LV‐Rasd1 transfection further decreased the level of UCP2, while the sh‐Rasd1 transfection reversed the inhibition of UCP2. Consequently, the OxyHb + sh‐Rasd1 + genipin group showed a lower UCP2 expression level than the OxyHb + sh‐Rasd1 group while the Rasd1 exhibited no statistical difference (Figure 10B–D). For ROS assay, the trend of fluorescence intensity change for DCFH‐DA was consistent with the trend of the above protein's expression change for Rasd1 or UCP2 (Figures 10E and 11F).

### 3.11 Rasd1 indirectly causes NCOA4‐mediated induced oligodendrocyte ferroptosis through neuronal UCP2 inhibition in vitro

Western blot and IF staining analysis demonstrated OxyHb resulted in OLs loss accompanied by GPX4 decreasing and NCOA4‐mediated ferritinophagy increasing. Rasd1 upregulation further exacerbated the loss of OLs, NCOA4‐mediated ferritinophagy increase, and GPX4 decrease. Rasd1 downregulation prevented the promoted effect of OxyHb‐induced OLs ferroptosis, meanwhile, the genipin treatment reversed the effect of sh‐Rasd1 for attenuating ferroptosis (Figure 11A–G). Primary OLs MDA and GSH assay results indicated that OxyHb resulted in the increase of lipid peroxidation levels, Rasd1 upregulation further aggravated lipid peroxidation, while Rasd1 downregulation declined it. Additionally, genipin administration reversed the effect of sh‐Rasd1 for anti‐lipid peroxidation (Figure 11H).

## 4 DISCUSSION

In the present study, we first found after 24 h post‐SAH, that Rasd1 reached a peak value and was mainly located in neurons, where the neuronal UCP2 decreased. OLs loss concomitant with oligodendrocytic NCOA4 increased in the favor of oligodendrocytic GPX4 and ferritin decreased. Then, Rasd1 overexpression increased neurological deficits, neuroinflammation and oxidative stress, activated glia, ultimately exacerbated WMI and ferritinophagy‐mediated OLs ferroptosis. But Rasd1 knockdown reversed the above effects. Additionally, Rasd1 was shown to inhibit the UCP2 activation, which can abate the harmful effects of Rasd1 on OLs ferroptosis through neurons. Overall, our findings demonstrated that Rasd1 caused ferroptosis of OLs participates in WMI of the SAH rat model, and functions by indirectly inducing ROS‐mediated ferritinophagy through neuronal UCP2 inhibition (Figure 11).

EBI refers to acute pathophysiologic effects due to subarachnoid bleeding and transient cerebral ischemia within the first 72 h.^29^ EBI has been shown to contribute to long‐term deficits after SAH.^30^, ^31^ Previous articles have reported ERS, autophagy impairment, glia activation, and mitochondrial dysfunction were involved in EBI through neuroinflammation and oxidative stress after SAH.^23^, ^32^, ^33^, ^34^ Researchers have consistently focused on neuronal injury and microglia activation post‐SAH.^35^, ^36^ Indeed, numerous studies currently shift the focus to the mechanism of WMI in EBI after SAH.^37^

WMI has been reported to include axonal injury, myelin loss, and oligodendrocyte progenitor cell differentiation disorders, it leads to cognitive, mental, perceptual, and motor deficits.^38^, ^39^, ^40^ Currently, most studies focus on oligodendrocyte progenitor cell maturation or death, polarized microglia or astrocytes mediate OLs loss, immunodeficiency reduces OLs, trauma directly damages OLs.^38^, ^41^, ^42^, ^43^ Despite some researches have reported on the OLs death mechanism in SCI, TBI, and ICH,^40^, ^44^ articles focusing on the Ols‐intrinsic death mechanism after SAH are lacking in the literature. In the current SAH study, we first showed the definitive evidence that mature Ols loss directly leads to WMI both in vivo and in vitro experiments. In vivo, from the perspective of molecular levels and structural levels, we used IF staining, Western blot, DTI, and TEM to unfold SAH insults to Ols. In vitro, oxyhemoglobin‐treated primary Ols greatest mimic SAH‐induced Ols morphological changes and the number varies, using Western blot and immunofluorescence. Both in vivo and in vitro results are exhibited visually.

Ferroptosis, an iron‐dependent programmed cell death, has been reported to contribute to multiple pathogenetic mechanisms in neurological disease.^45^ The abnormal iron accumulation could catalyze oxygen radicals via the Fenton reaction, which induces oxidative stress injury and eventually develops into irreversible neurological damage.^46^ Overloaded iron derives from exocytic iron transported by membrane receptors and intracellular iron degraded by ferritin.^47^ Accordingly, our group has directed its interest to study the relationship between iron metabolism and EBI post‐SAH. We and others have revealed DMT1‐mediated iron transportation and ferritinophagy‐mediated ferritin degradation, which could lead to neuronal ferroptosis and contribute to EBI post‐SAH.^13^, ^48^ Furthermore, to our knowledge this is the first study to focus on Ols ferroptosis after SAH, which triggers WMI, thus involving EBI complications. As previously described, intracellular ferritin has been pinpointed as a source of free iron in mature Ols.^24^ Our in vivo and in vitro studies have demonstrated that NCOA4‐mediated ferritinophagy participated in Ols ferroptosis, which directly induces WMI.

Rasd1, found predominantly in the brain, has played a crucial role in circadian control, metabolic diseases, neurotoxicity, and anxiety disorders. As previous findings demonstrated, Rasd1 almost functions in neurons and it is downstream of nNOS‐CAPON and is involved in ERK pathway inhibition and iron homeostasis. Briefly, Rasd1 respectively suppresses ERK‐CREB‐BDNF pathway and PAP7‐DMT1 pathway.^19^, ^49^ Thus, Rasd1mediates neurotoxicity through excess neuronal iron uptake and inhibiting BDNF protective capacities in neurons. Owing to this, our study revealed the role of Rasd1 in WMI rather than neuronal injury, where we have found that Rasd1 indirectly resulted in Ols iron overload through neuron‐oligodendrocyte communication. We have identified this interaction using co‐culture of primary neurons and Ols in vitro study. After LV‐Rasd1 transfected neurons, neurons produced ROS accompanied by the occurrence of ferritinophagy in Ols. Besides, our in vivo results found Rasd1 deficiency abated the neurological deficits, cognitive impairments, glia activation, neuroinflammatory response, and oxidative stress due to SAH. Meanwhile, independently, it has been confirmed inflammatory cytokines could activate IL‐6 / STAT3 signaling to inhibit GPX4 expression, which induces ferroptosis.^50^ Taking all the together, these results suggested a tight connection between Rasd1 with Ols ferroptosis, WMI, EBI post‐SAH.

UCP2, which is found to be located in the mitochondrial inner membrane, has been implicated in a critical role in modulating ROS homeostasis^51^ as well as metabolism and immunity.^52^ Previous research revealed UCP2 exerted neuroprotection in multiple central nervous system injuries.^53^, ^54^ UCP2 has been reported as a downstream target of PKA signaling and the AMPK pathway, functioning by attenuated oxidative stress and neuroinflammation.^23^, ^34^ Recently, there are few controversial studies indicating that ROS could trigger the NCOA4‐mediated ferritinophagy and lead to ferroptosis.^55^, ^56^ Given the above findings, our study has explored whether neuronal UCP2 could negatively modulate NCOA4‐mediated ferritinophagy of Ols through neuron‐oligodendrocyte communication. Genipin, a UCP2 inhibitor, has been applied in in‐vitro experiments. Finally, our results indicated that genipin elevated NCOA4 expression, increased ferritin degradation, decreased GPX4 levels, and aggravated Ols loss. Therefore, the study confirmed our speculation.

In this study, our work revealed the role of Rasd1 in triggering Ols ferroptosis and exacerbating WMI after SAH by neuron‐oligodendrocyte communication. In brief, Rasd1 inhibits neuronal UCP2, resulting in NCOA4‐mediated ferritinophagy and declined GPX4 expression in Ols.

There are also some limitations, which will need to be addressed. Firstly, although it was reported Rasd1 could inhibit cAMP/PKA/CREB, and UCP2 could be downstream of PKA/CREB, and our results confirmed Rasd1 could inhibit UCP2 meanwhile, we cannot rule out the possibility that Rasd1 may exhibit UCP2 inhibition via other pathways. Then, glia activation was reported that could be stimulated by ROS or inflammatory cytokines,^28^ and we observed this phenomenon; however, the exact mechanism needs to be deeply explored. We need more clear evidence about the above two aspects in future studies to further elucidate unknown potential mechanisms in EBI post‐SAH.

## 5 CONCLUSION

As a conclusion, our study indicated that SAH‐induced upregulation of Rasd1 could trigger WMI through neuronal Rasd1/UCP2‐oligodendrocytic NCOA4/ferritin axis in a form of neuron‐oligodendrocyte communication.

## AUTHOR CONTRIBUTIONS

WQF and ZHH were responsible for the experiment design, WQF, XDC, JHT, and YRM performed the experiments, SZC and DQX were responsible for all data analyzed, WQF, HBL, XLY, and TMW were responsible for the manuscript writing. All authors approved the final manuscript.

## CONFLICT OF INTEREST STATEMENT

The authors declare no conflict of interest.

## Supporting information


Appendix S1

## Data Availability

All data generated in this study have been included in this article.
