# Supplementary material for: Rasd1 is involved in white matter injury through neuron‐oligodendrocyte communication after subarachnoid hemorrhage
Source: CNS Neurosci Ther. 2023 Sep 22;30(3):e14452. doi: 10.1111/cns.14452 (PMC10916428; doi:10.1111/cns.14452)
Supplement: Supplementary file 1 — Appendix S1 [file CNS-30-e14452-s001.zip › experimental groups.docx]

**Experiment design.** Experiment 1: Rasd1 time course expression changes and cellular localization after SAH. Experiment 2: Confirm the best lentivirus dosage through the effects of different Rasd1 levels on neurological function and mortality in SAH rats. Experiment 3: In vivo study, confirm the role of Rasd1 involved in white matter injury after SAH from different aspects in vivo study. Experiment 4: In vitro study, confirm the molecular mechanism of Rasd1 mediated oligodendrocytes ferroptosis through neuron-oligodendrocyte communication.
