# Supplementary figures and images for: Rasd1 is involved in white matter injury through neuron‐oligodendrocyte communication after subarachnoid hemorrhage
Source: CNS Neurosci Ther. 2023 Sep 22;30(3):e14452. doi: 10.1111/cns.14452 (PMC10916428; doi:10.1111/cns.14452)

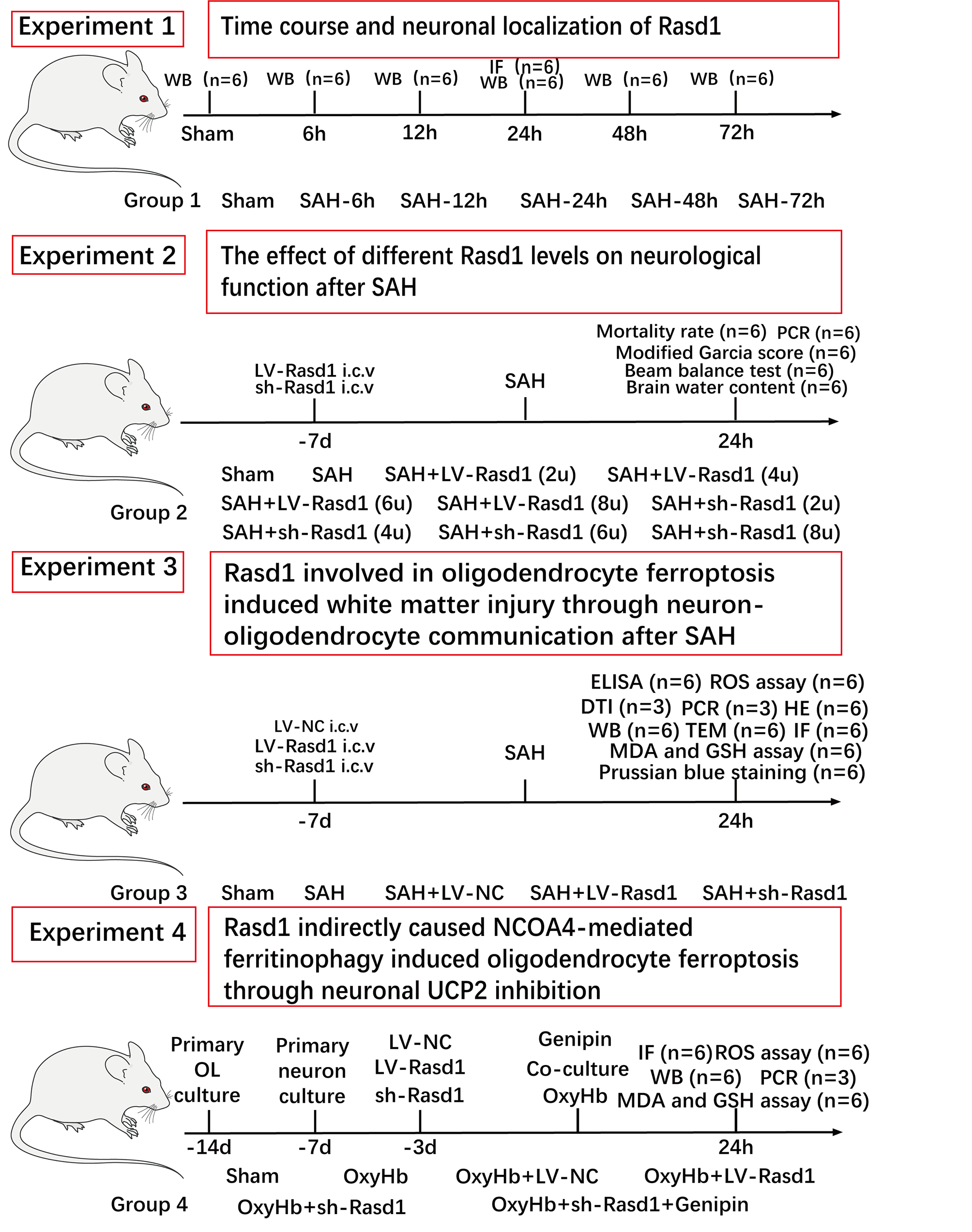

Supplement: Supplementary file 1 — Appendix S1 [file CNS-30-e14452-s001.zip › experimental groups.tif]
